# Racial and Ethnic Disparities in Drug Overdose Deaths in the US During the COVID-19 Pandemic

**DOI:** 10.1001/jamanetworkopen.2022.32314

**Published:** 2022-09-20

**Authors:** Beth Han, Emily B. Einstein, Christopher M. Jones, Jessica Cotto, Wilson M. Compton, Nora D. Volkow

**Affiliations:** 1 National Institute on Drug Abuse, National Institutes of Health, Bethesda, Maryland; 2 National Center for Injury Prevention and Control, Centers for Disease Control and Prevention, Atlanta, Georgia

## Abstract

This cross-sectional study describes the nationwide trends in drug overdose mortality during the COVID-19 pandemic by age, sex, and race and ethnicity.

## Introduction

Recent reports highlighted racial and ethnic differences in US drug overdose deaths from 1999 to 2020.^1,2^ Overdose deaths increased 37.2% from February 2020 to August 2021 and were predominantly associated with synthetic opioids other than methadone (primarily fentanyl or analogs) and methamphetamine.^3,4^ Yet data are lacking regarding racial and ethnic disparities in overdose death rates within specific sex-age combinations before and during the COVID-19 pandemic (since March 2020).

## Methods

This study used publicly available deidentified data and was deemed exempt from review by the National Institutes of Health Institutional Review Board. We followed the STROBE reporting guideline.

Semiannual drug overdose death data were obtained from the National Vital Statistics System Multiple Cause-of-Death final (March 2018-December 2020) and provisional files (January-August 2021). These deaths were identified using *International Statistical Classification of Diseases and Related Health Problems, Tenth Revision,* codes X40–X44 (unintentional) and Y10-14 (undetermined intent). Deaths involving synthetic opioids other than methadone (largely fentanyl or analogs) were identified using code T40.4. Deaths involving psychostimulants with abuse potential other than cocaine (largely methamphetamine) were identified using code T43.6.

Age-adjusted, sex- and race and ethnicity–specific overdose death rates were computed for overall drug, fentanyl, and methamphetamine with or without fentanyl among individuals aged 15 to 34 years and 35 to 64 years from March 2018 to August 2021. Joinpoint Regression Program, version 4.8.01 tested for significant changes in nonlinear trends using bayesian information criterion. Two-sided *P* < .05 was considered significant.

## Results

Among individuals in the US aged 15 to 34 years from March 2018 to August 2021, age-adjusted overdose death rates involving any drug, fentanyl, and methamphetamine with or without fentanyl increased overall (Figure; Table). During March to August 2021, non-Hispanic American Indian or Alaska Native (hereafter American Indian or Alaska Native) men had the highest rates per 100 000 overall involving any drug (42.0; 95% CI, 35.5-48.4 per 100 000), fentanyl (30.2; 95% CI, 24.7-35.7), and methamphetamine without fentanyl (6.0; 95% CI, 3.6-8.5). American Indian or Alaska Native men (9.2; 95% CI, 6.1-12.2) and women (8.0; 95% CI, 5.1-10.9) and non-Hispanic White (hereafter White) men (6.7; 95% CI, 6.4-7.0) had the highest rates involving methamphetamine with fentanyl.

**Figure.**
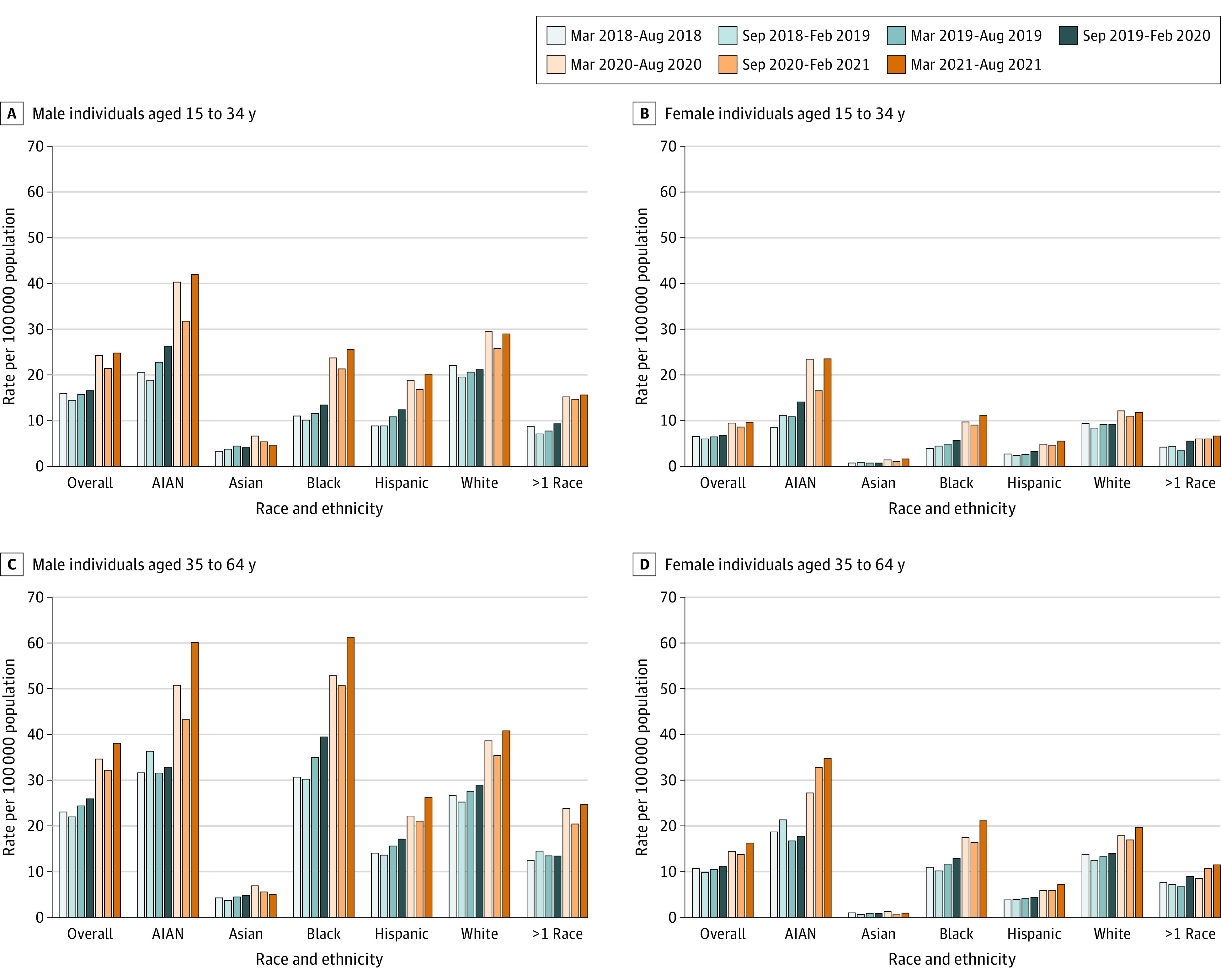
Age-Adjusted Drug Overdose Death Rates Among US Individuals by Age, Sex, and Race and Ethnicity Before and During the COVID-19 Pandemic March-August 2020 indicates the start of the COVID-19 pandemic. AIAN indicates American Indian or Alaska Native.

**Table.** National Trends in Age-Adjusted Drug Overdose Death Rates for Deaths Involving Any Drug, Synthetic Opioids Other Than Methadone (Fentanyl), and Psychostimulants Other Than Cocaine (Methamphetamine) With or Without Fentanyl, by Age, Sex, and Race/Ethnicity

	Drug overdose death^a^					Fentanyl^b^					Methamphetamine with Fentanyl^b^^,^^c^					Methamphetamine without Fentanyl^b^^,^^c^				
	Age-adjusted death rates per 100 000			Mar-Aug 2018 to Mar-Aug 2021 ASPC	*P* value for ASPC	Age-adjusted death rates per 100 000			Mar-Aug 2018 to Mar-Aug 2021 ASPC	*P* value for ASPC	Age-adjusted death rates per 100 000			Mar-Aug 2018 to Mar-Aug 2021 ASPC	*P* value for ASPC	Age-adjusted death rates per 100 000			Mar-Aug 2018 to Mar-Aug 2021 ASPC	*P* value for ASPC
	Mar- Aug 2018	Mar- Aug 2020	Mar- Aug 2021			Mar-Aug 2018^d^	Mar-Aug 2020	Mar-Aug 2021			Mar-Aug 2018^**d**^	Mar-Aug 2020^**d**^	Mar- Aug 2021^**d**^			Mar-Aug 2018^**d**^	Mar-Aug 2020^**d**^	Mar-Aug 2021^**d**^		
**Age 15-34 y**																				
Men	16.0	24.2	24.8	10.2	.002	9.7	18.5	19.8	15.9	*<*.001	1.1	3.7	5.2	32.8	*<*.001	1.7	2.1	2.1	2.3	.01
Race and ethnicity^e^																				
AIAN	20.5	40.3	42.0	15.0	.001	8.3	28.3	30.2	27.4	*<*.001			9.2	NA^f^		5.6	7.0	6.0	NA^f^	
Asian	3.3	6.6	4.6	8.5	.05	1.6	4.9	3.3	16.1	.02				NA^f^					NA^f^	
Black	11.0	23.7	25.5	19.4	.001	6.3	19.0	20.5	26.1	.001	0.6	2.8	3.6	1.8	*<*.001	1.1	1.3	1.9	5.6	.07
Hispanic	8.8	18.8	20.1	16.8	*<*.001	4.4	13.7	16.1	25.9	*<*.001	0.4	2.3	3.7	47.6	*<*.001	1.9	1.9	1.8	2.2	.26
White	22.1	29.5	29.0	7.1	.007	13.9	22.5	23.2	11.9	.002	1.6	4.8	6.7	29.4	*<*.001	2.1	2.5	2.4	1.7	.07
>1 race	8.7	15.2	15.6	14.9	.009	4.3	12.0	12.3	24.5	.003		3.2	3.8	NA^f^				1.8	NA^f^	
Women	6.5	9.5	9.6	9.1	.002	3.9	6.9	7.6	14.8	*<*.001	0.6	1.6	2.1	26.7	*<*.001	1.7	2.1	2.1	−0.2	.91
Race and ethnicity^e^																				
AIAN	8.5	23.4	23.5	16.9	*<*.001		14.6	18.9	32.2	.02			8.0	NA^f^		5.6	7.0	6.0	NA^f^	
Asian	0.8	1.4	1.6	12.8	.006		0.9	1.0	NA^f^					NA^f^					NA^f^	
Black	3.9	9.7	11.2	21.1	*<*.0001	2.5	7.2	9.1	26.9	*<*.001		1.0	1.4	NA^f^		1.1	1.3	1.9	NA^f^	
Hispanic	2.7	4.9	5.5	16.2	.001	1.3	3.4	4.2	25.2	*<*.001		0.7	1.1	NA^f^		1.9	1.9	1.8	2.1	.59
White	9.4	12.1	11.8	6.1	.005	5.7	8.9	9.3	11.2	*<*.001	0.9	2.3	2.9	23.3	*<*.001	2.1	2.5	2.4	−2.0	.004
>1 race	4.2	6.0	6.7	9.8	.006	2.0	4.1	4.8	15.5	*<*.001				NA^f^				1.8	NA^f^	
**Age 35-64 y**																				
Men	23.0	34.6	38.0	10.3	*<*.001	11.5	22.3	25.9	16.8	*<*.001	1.3	4.7	7.4	37.6	*<*.001	3.9	5.5	6.0	7.6	*<*.001
Race and ethnicity^e^																				
AIAN	31.6	50.7	60.0	10.5	.003	9.5	21.7	30.9	22.0	.02		5.4	12.6	NA^f^		12.6	17.2	22.9	4.9	.06
Asian	4.2	6.9	5.0	7.0	.08	1.1	2.6	2.3	17.5	.21			0.6	NA^f^		1.2	1.7	1.7	6.4	.007
Black	30.6	52.8	61.2	13.8	*<*.001	16.3	35.7	43.3	19.4	*<*.001	0.6	3.6	5.9	46.7	*<*.001	2.6	4.3	5.0	13.7	*<*.001
Hispanic	14.0	22.1	26.2	12.1	*<*.001	6.2	13.2	17.1	20.4	*<*.001	0.4	2.0	4.3	54.0	*<*.001	3.3	4.8	5.0	8.1	*<*.001
White	26.6	38.5	40.7	9.1	*<*.001	13.7	25.3	28.0	15.2	*<*.001	1.8	6.2	9.5	34.9	*<*.001	4.4	6.1	6.6	6.9	*<*.001
>1 race	12.4	23.8	24.6	13.1	.002	3.6	11.0	15.3	30.8	*<*.001		4.0	6.4	NA^f^		5.9	10.5	6.9	5.5	.01
Women	10.7	14.4	16.2	8.9	*<*.001	4.3	8.1	10.1	17.8	*<*.001	0.5	1.9	3.0	37.1	*<*.001	1.5	2.1	2.3	7.9	*<*.001
Race and ethnicity^e^																				
AIAN	18.7	27.2	34.8	13.1	.008		12.8	17.5	32.9	.004		5.7	9.4	NA^f^		8.8	8.3	11.6	6.2	.37
Asian	1.0	1.3	0.9	0.8	.92		0.5	0.5	NA^f^					NA^f^					NA^f^	
Black	10.9	17.4	21.1	13.3	*<*.001	5.1	10.6	14.3	20.3	*<*.001		0.9	1.4	34.4	*<*.001	0.6	1.2	1.3	12.8	*<*.001
Hispanic	3.8	5.9	7.2	12.1	*<*.001	1.2	3.1	4.2	25.0	*<*.001		0.6	1.0	NA^f^		0.8	1.2	1.3	8.8	*<*.001
White	13.8	17.8	19.7	8.0	*<*.001	5.6	10.1	12.2	16.4	*<*.001	0.8	2.7	4.3	36.1	*<*.001	1.9	2.5	2.9	7.7	*<*.001
>1 race	7.6	8.5	11.5	9.3	.001		3.7	6.9	27.2	*<*.001			3.2	NA^f^		2.9	3.3		NA^f^	

Abbreviations: AIAN, American Indian or Alaska Native; ASPC, average semiannual percentage change; *ICD-10, International Statistical Classification of Diseases and Related Health Problems, Tenth Revision*; NA, not available.

^a^ *ICD-10* codes X40-44 (unintentional) and Y10-14 (undetermined intent).

^b^ *ICD-10* code T40.4: synthetic opioids other than methadone, including fentanyl or analogs.

^c^ *ICD-10* code T43.6: psychostimulants with abuse potential other than cocaine, largely methamphetamine.

^d^ Missing values represent suppressed estimates. To estimate age-adjusted death rates simultaneously by age, sex, and race and ethnicity, age 35 years was used as the threshold between the 2 age categories for sufficient overdose deaths in each examined subgroup.

^e^ Race and ethnicity data were obtained from the National Vital Statistics System files and included the following recoded categories: AIAN, Asian, Black or African American, Hispanic, Native Hawaiian or Other Pacific Islander, not Hispanic, White, more than 1 race, and not stated. Age-adjusted drug overdose death rates for Native Hawaiians or Other Pacific Islander individuals were not reported because most were suppressed, and rates for those with unknown ethnicity were not estimated due to the lack of precise denominators.

^f^ NA indicates insufficient valid data points for joinpoint analyses. Because of space limitations, only age-adjusted semiannual mortality rates for March to August 2018, March to August 2020 (beginning of the COVID-19 pandemic), and March to August 2021 are displayed. Trend analyses were based on all 7 of the semiannual periods between March to August 2018 and March to August 2021, and nondisplayed data are available on request.

Among individuals aged 35 to 64 years from March 2018 to August 2021 (Figure; Table), age-adjusted overdose death rates per 100 000 increased overall for deaths involving any drug, fentanyl, and methamphetamine with or without fentanyl among most subgroups. During March to August 2021, overall drug overdose rates were highest among non-Hispanic Black or African American (hereafter Black) men (61.2; 95% CI, 59.4-62.9) and American Indian or Alaska Native men (60.0; 95% CI, 52.8-67.2), and fentanyl-involved death rates were highest among Black men (43.3; 95% CI, 41.8-44.8). Rates involving methamphetamine with fentanyl were highest among American Indian or Alaska Native men (12.6; 95% CI, 9.2-16.0) and women (9.4; 95% CI, 6.5-12.3) and White men (9.5; 95% CI, 9.1-9.8). Rates involving methamphetamine without fentanyl were highest among American Indian or Alaska Native men (22.9; 95% CI, 18.4-27.4).

## Discussion

Drug overdose mortality continued to increase from March to August 2018 through the COVID-19 pandemic for almost all examined subgroups, with increases primarily attributed to fentanyl/analogs and methamphetamine. By March to August 2021, across sexes, the highest drug overdose death rates were among American Indian or Alaska Native men aged 15 to 34 years and Black and American Indian or Alaska Native men aged 35 to 64 years. Within every age and racial and ethnic group, rates were higher in men; for women, and the highest drug overdose death rates were among American Indian or Alaska Native individuals. Limitations of this study are that overdose deaths may be underestimated because of the use of 2021 provisional data^3^ and that racial or ethnic identification may be misclassified, especially for American Indian or Alaska Native people.^5^

Results underscore the urgency of expanding prevention, treatment, and harm reduction interventions tailored to specific populations, especially American Indian or Alaska Native and Black populations, given long-standing structural racism and inequities in accessing these services.^4,6^ Findings also suggest the urgent need for education on dangers of methamphetamine and fentanyl. Reducing overdose mortality disparities may include expanding access to naloxone, fentanyl test strips, and treatments for substance use disorders to disproportionately affected populations.
